# Two-Year Follow-Up after Contact Force Sensing Radiofrequency Catheter and Second-Generation Cryoballoon Ablation for Paroxysmal Atrial Fibrillation: A Comparative Single Centre Study

**DOI:** 10.1155/2016/6495753

**Published:** 2016-05-22

**Authors:** Attila Kardos, Zsuzsanna Kis, Zoltan Som, Zsofia Nagy, Csaba Foldesi

**Affiliations:** ^1^Department of Cardiac Electrophysiology, Gottsegen Gyorgy National Institute of Cardiology, Haller Street 29, Budapest 1096, Hungary; ^2^Department of Clinical Electrophysiology, Erasmus Medical Center, 's-Gravendijkwal 230, Postbus 2040, 3015 CE Rotterdam, Netherlands

## Abstract

*Background*. There are little comparative data on catheter ablation of paroxysmal atrial fibrillation (AF) using the contact force radiofrequency (CF-RF) catheter versus the second-generation cryoballoon (CB2).* Methods and results*. This is a single center, retrospective, nonrandomized study of 98 patients with symptomatic, drug-refractory paroxysmal AF who underwent their first PVI ablation using either the CB2 (*n* = 40) or CF-RF (*n* = 58). The mean age was 60 years with 63% men, a mean LA size of 42 mm. The procedure duration (74 ± 17 versus 120 ± 49 minutes *p* < 0.05) was shorter for CB2 group; the fluoroscopy time (14 ± 17 versus 16 ± 5 minutes, *p* = 0.45) was similar. Complete PVI was achieved in 96% of patients with RF-CF and 98% with CB2. Phrenic nerve palsies (2 transient and 1 persistent) occurred exclusively in the CB2 group and 1 severe, nonlethal complication (pericardial tamponade) occurred in the CF-RF group. At 24-month follow-up, the success rate, defined as freedom from AF/atrial tachycardia (AT) after a single procedure without antiarrhythmic drug, was comparable in CF-RF group and CB2 group (65.5% versus 67%, resp., log rank *p* = 0.54).* Conclusion*. Both the CB2 and the RF-CF ablation appeared safe; the success rate at 2 years was comparable between both technologies.

## 1. Introduction

In transcatheter ablation of pulmonary veins (PVs), pulmonary vein isolation (PVI) has been established as an effective therapeutic option for patients with symptomatic drug-refractory paroxysmal atrial fibrillation (AF). PVI can be successfully achieved with different energy sources; radiofrequency ablation (RF) and cryoballoon (CB) ablation are the most frequently used technologies [1–6]. RF ablation by means of a focal-tip catheter in combination with a 3D mapping system is still the standard technique performed worldwide. With continuous improvements in catheter technology, such as contact force radiofrequency (CF-RF), additional clinical benefit during PVI is reported when compared to non-CF technology in recent prospective, nonrandomized trials. Marijon et al. reported a potential benefit of real-time contact force sensing technology (compared to conventional irrigated radiofrequency ablation catheter), in reducing AF recurrence during the first year after PVI in 60 patients, Jarman et al. reported the results of a total of 600 ablation procedures (200 using CF-RF), the mean follow-up was 12 months, and the use of force sensing catheters independently predicted clinical success in paroxysmal AF patients [7, 8]. Operator experience is a determinant of outcome after PVI even in high-volume centres [9]. In recent years, the use of novel balloon based technologies such as cryoballoon ablation has emerged as an alternative approach to point-by-point technology [5, 6]. CB technology might offer more reproducible and standardizable procedures by simplifying the ablation itself. There are certain practical advantages, such as reduced pain during ablation, improved catheter stability due to catheter-tissue adhesion, and ability to rapidly create circumferential lesions. Although it is clear that the patients benefit more from an experienced cryoballoon operator, Mikhaylov et al. showed that, in low-volume centres, where operators have experience in simple ablation procedures, CB is a safe procedure with success rates comparable to those obtained in higher-volume AF centres. This underlines that CB has a fast and reproducible learning curve in both high- and low-volume AF ablation centres [10]. The first-generation CB was compared to standard RF ablation by Mugnai et al., who reported similar AF recurrence and AF episode burden after pulmonary vein isolation using conventional irrigated radiofrequency energy versus the first-generation cryoablation. Freedom from AF was reached in 63.5% of patients treated with cryothermia and 57.3% of patients treated with RF at 23-month follow-up [11]. The prospective FreezeAF trial demonstrated noninferiority of CB ablation versus RF ablation for treating patients with paroxysmal AF. The primary endpoint (freedom from atrial arrhythmia) at 12 months was achieved by 70.7% with RF and 73.6% with CB (multiple procedure success), including 31 redo procedures in each group (19.5% of RF versus 19.9% of CB; *p* = 0.933) [12].

Recently, the second-generation CB (CB2) has been introduced featuring homogeneous cooling of the entire frontal surface with increased refrigerant flow [13]. Significantly improved clinical outcome was demonstrated by Fürnkranz et al. (83.6% versus 63.9% of arrhythmia-free survival) upon using the second-generation CB after a single procedure without AAD therapy after 1 year when compared to the first-generation CB [14]. A recently published multicentre, retrospective study of more than 1100 patients suggests that freedom from atrial fibrillation following PVI using the second-generation CB is superior to open irrigated, non-force sensing RF based ablation at 12 months following a single procedure [15].

To the best of our knowledge, published data on comparison of medium- and long-term results between new generation CB and conventional manual CF-RF ablation are limited. The aim of this study was to compare the efficacy and safety of the new cryoballoon ablation (CB2) with the CF-RF approach in the treatment of paroxysmal AF.

## 2. Methods

This was a nonrandomized, retrospective, single centre study. From September 2012 to December 2013, 98 patients with drug resistant paroxysmal atrial fibrillation (defined as self-terminated, documented episodes lasting <7 days) who underwent initial PVI using either radiofrequency energy with contact force sensing ablation catheter (ThermoCool SmartTouch, Biosense Webster Inc., Diamond Bar, CA, USA; *n* = 58) or the second-generation cryoballoon (Arctic Front Advance, Medtronic, Minneapolis, MN, USA; *n* = 40) were enrolled. Written informed consent was obtained from all the patients. Before the procedure, all patients underwent a 2-dimensional transthoracic echocardiography to assess left ventricular ejection fraction and atrial dimensions and to rule out any structural and/or valvular disease. A transesophageal echocardiography was performed the day before ablation to analyze left atrial (LA) and PV anatomy and to rule out intracardiac thrombus formation. Antiarrhythmic medications were not stopped before the procedure.

All patients received anticoagulation therapy during the 4 weeks before and for at least 3 months after the index procedure. Periprocedurally, administration of the anticoagulation therapy was uninterrupted, regardless of the selected drug.

RF ablation instances were performed as follows.

Two nonsteerable long sheaths (St. Jude Medical, St Paul, MN, USA) were introduced into LA using a double transseptal puncture technique. An initial intravenous bolus of heparin (100 IU/kg) was followed by extra boluses to maintain an activated clotting time (ACT) > 300 s. An electroanatomic map of the LA was performed using CARTO system (Biosense Webster Inc., Diamond Bar, CA, USA) with a circumferential mapping catheter (LassoNav*™*, Biosense Webster Inc., Diamond Bar, CA, USA). Antral PVI was performed with open irrigated tip 3.5 mm force sensing ablation catheter (Navistar Thermocool SmartTouch, Biosense Webster Inc., Diamond Bar, CA, USA) in a power-controlled mode with a power limit of 35 W and at a maximum temperature of 48°C. Power was reduced to 25 W during ablation of LA posterior wall to prevent esophageal injury. The PVs were isolated by a circumferential lesion set. The endpoint was elimination of local bipolar electrogram during ablation with 20–40 s lesions. The VisiTag*™* module of CARTO 3 had been used in patients since January 2013; the settings during LA ablation was a modification of the settings reported by Lin et al. [16]:

- A minimum time of 20 seconds.
- A maximum range of 4 mm.
- A force over time of 50%.
- A minimum force of 6 g.

The desired minimum CF was 10 g for lesion formation; upper limit defined was 50 g force to avoid perforation.

CB ablation instances were performed as follows.

A single transseptal puncture was achieved under fluoroscopic guidance. After gaining the LA access, a 100 UI/kg heparin intravenous bolus was given, as well as extra boluses to maintain ACT > 300 s. A 0.32 Fr exchange wire (Emerald*™* Cordis, Johnson and Johnson, Diamond Bar, CA, USA) was advanced in the left superior PV and a steerable 15 Fr over-the-wire sheath (FlexCath Advance*™*, Medtronic, Minneapolis, MN, USA) was positioned in the left atrium. A circular mapping catheter (Achieve*™*, Medtronic, Minneapolis, MN, USA) together with a second-generation 28 mm double-walled CB (Arctic Front Advance, Medtronic, Minneapolis, MN, USA) was then advanced and positioned in the PV ostium of each vein. Vessel occlusion was evaluated according to a semiquantitative grading ranging from grade 0 (very poor occlusion) to grade 4 (perfect occlusion) after dye injection; optimal vessel occlusion was deemed as total contrast retention with no backflow in the atrium. For each vein, cryoablation consisted of ≥1 applications lasting 4 min. Phrenic nerve function was monitored during right superior vein cryoablation by fluoroscopic assessment of diaphragm movement during spontaneous breathing.

All procedures were performed under deep sedation utilizing fractionated intravenous bolus of midazolam and fentanyl or continuous infusion of propofol with preservation of spontaneous breathing and continuous monitoring of oxygen saturation.

PV isolation was guided by circular mapping catheter (LassoNav, Biosense Webster Inc., Diamond Bar, CA, USA, and Achieve, Medtronic, Minneapolis, MN, USA) in both groups. Once entrance block was confirmed, pacing from all the bipoles on the circular mapping catheter was used to assess exit block by using a pacing stimulus of 10 mA at 2 ms. Evidence for PV capture without conduction to LA was necessary to prove exit block. The procedures were stopped immediately after initial isolation; adenosine was not administered.

Postprocedural anticoagulation was started after sheath removal with low molecular weight heparin, or NOAC. Anticoagulation after the procedure was continued for at least three months or longer depending on the individual CHA_2_DS_2_-VASc score. Antiarrhythmic drugs were continued for 3 months after ablation and then were stopped.

### 2.1. Patient-Follow-Up

Clinical follow-up consisted of physical examinations, 12-lead ECG, and 24-hour Holter recording performed at 3, 6, and 12 months after ablation and every 6 months after the first year. At 9, 15, and 21 months, patients were asked about their AF symptoms with standardized questionnaires by telephone. We directed patients to check their pulse rate and rhythm at least once a day and to visit the outpatient clinic if they experienced relapse of AF.

A blanking period of 3 months was considered for the study. All documented episodes of atrial tachyarrhythmias lasting 30 seconds and any symptoms suggesting AF were considered as recurrence. Success rate was defined as the percentage of patients who did not have any documented AF episode or any symptoms suggesting AF recurrence after the blanking period within the follow-up period.

### 2.2. Statistical Analysis

Continuous measures are expressed as the mean value ± standard deviation and were analyzed with the two-sided *t*-test after testing for normal distribution with the method of Kolmogorov and Smirnov. *χ* ^2^ test was used for between-groups comparison. Kaplan-Meier event-free survival analysis was conducted to assess the cumulative freedom from recurrence. A *p* value <0.05 was considered statistically significant.

## 3. Results

Starting in September 2012, a total of 98 patients were included in the study.

Baseline characteristics are shown in Table 1.

Procedure time (from venous puncture to removal of sheaths) was significantly shorter in the CB2 group (74 ± 17 minutes versus 120 ± 49, *p* < 0.05); fluoroscopy time was similar in both groups (14 ± 17 versus 16 ± 5 minutes, *p* = 0.45). Complete PVI was achieved in 98% (39 of 40) of cryoballoon-treated patients and in 96% (56 of 58) of RF-treated patients. The 28 mm cryoballoon was used during all procedures without touch-ups. Three left common PVs (LCPVs) were identified in the 40 patients. The mean number of CB applications per PV was 1.5 ± 0.8 for the LSPV, 1.3 ± 0.6 for the LIPV, 1.5 ± 0.8 for the RSPV, 1.7 ± 0.9 for the RIPV, and 2.0 ± 1.3 for the LCPV, respectively.

The minimum balloon temperatures (as marker of balloon-tissue contact) measured were lower in the inferior PVs: LSPV: −49.5 ± 6°C versus LIPV: −44.6 ± 7°C (*p* < 0.05) and RSPV: −50 ± 7°C versus RIPV: −41 ± 10°C (*p* < 0.001), respectively.

Real-time catheter-tissue contact force and force time integral (FTI) were continuously monitored in 51 out of 58 CF-RF patients.

**Figure 1:**
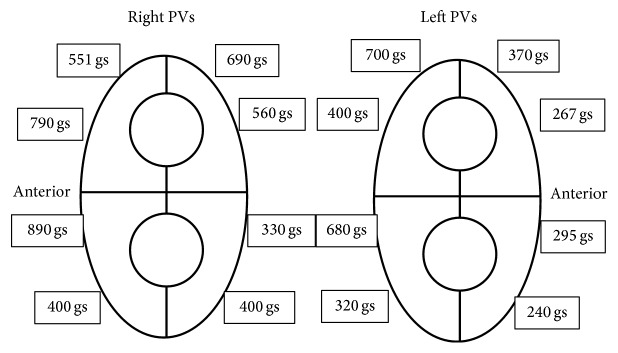
Distribution of mean force time integral (FTI) values (gs) per PV-quadrants in the CF-RF group.

Significantly lower FTI values were detected on the anterior and inferior aspects of the left pulmonary veins and the posteroinferior region of the right inferior pulmonary vein as compared to the other PV regions using nonsteerable sheaths (Figure 1).

**Figure 2:**
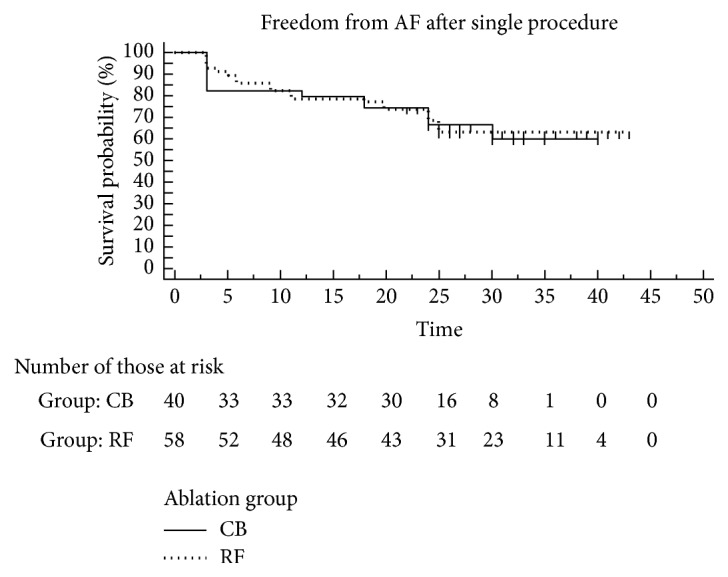
Kaplan-Meier estimation of the time to AF recurrence after single ablation in the CF-RF and CB ablation groups (CB group: solid line; RF group: dotted line).

All patients completed the 12-month and the extended 24-month follow-up. At 1 year, 77.5% (45/58) in the CF-RF group and 80% (32/40) in the CB2 group were free from recurrent AF/AT, while at 24 months 65.6% (38/58) in the CF-RF group and 67.5% (27/40) in the CB2 group off AAD remained in sinus rhythm without statistical significance (Figure 2).

After index PVI, the previously ineffective AAD was started in all patients experiencing recurrence. A total of 22 patients unresponsive to AAD (15 pts from CF-RF and 7 pts from CB group) suffering from AF recurrence underwent a repeat ablation 13.3 ± 7.8 months after initial PVI. All repeat procedures were performed by the means of RF energy. In the CF-RF group, among 60 PVs, 37 (61%) showed conduction gaps in 15 patients (2.5 per patient), whereas in the CB2 group, among 28 veins, 10 (35%) showed a PV reconnection in 6 patients (1.4 per patient) (*p* = 0.01). In one CB2 patient, all the PVs were isolated despite of documented symptomatic paroxysmal atrial fibrillation episodes; non-PV foci were ablated.

Reconnection rates per vein in the CF-RF group were as follows: LSPV: 53% (8/15), LIPV: 66% (10/15), RSPV: 40% (6/15), and RIPV: 87% (13/15).

The inferior pulmonary veins (the left and right inferior PVs) were frequently reconnected in the CB2 group (86% 12/14 veins), while conduction gaps could be documented in the right and left superior PV only in one patient.

Procedural complication rate was low. One pericardial tamponade treated by pericardiocentesis in the CF-RF group was detected, with 3 phrenic nerve palsies in the CB2 group, two resolved completely before hospital discharge, and the third also did resolve after 12 months.

## 4. Discussion

As far as we know, these are the first published data with the longest follow-up to compare the feasibility and efficacy of the second-generation cryoballoon ablation in circumferential PV isolation with contact force sensing RF ablation in paroxysmal atrial fibrillation after 2 years. Though the results of this single centre, retrospective study should be interpreted carefully, several findings are noteworthy.

First, in our study no differences of medium- and long-term success rates were found between both CF-RF and CB2 groups (76.5% versus 81% at 12 months and 65.5% and 67.5% at 24 months). Our results are similar to those of the SMART-AF trial, where the 12-month success rate was 74% with CF-RF catheters [17] while several studies reported 80% or even higher clinical success with the new generation cryoballoon at 1 year [14, 18]. In the recently published FIRE and ICE multicentre randomized study where the mean duration of follow-up was 1.5 years, the reported recurrence rates were 34.6% and 35.9% in the cryoballoon and in the radiofrequency group. Although the representation of the most advanced generation catheters was not equal in this study (first-generation CB (24%) and CB2 (76%) in the CB group and non-CF-RF (76%) and CF-RF (24%) in the RF group), no significant difference was found between the catheters used in terms of efficacy and safety [19].

The use of nonsteerable long sheath might have influenced the success rate in the CF-RF group in our study. The use of steerable sheath theoretically would improve the outcome of CF-RF ablation; these devices were designed to improve access to and contact with ablation target sites. One prospective, randomized, controlled study demonstrates that AF catheter ablation using a steerable transseptal sheath is associated with a significantly better rhythm control compared with an ablation with a nonsteerable sheath. Single procedure outcome has improved by 20% [20].

The improvements in cryoballoon cooling homogeneity found in Arctic Front Advance may address some issues that impeded long-term success of cryoballoon ablation as reported by several authors [13–15]. In fact, the CB2 might achieve significantly lower temperatures and faster isolation times in comparison with the first-generation device [14]. We aimed for single 4-min-duration freeze-thaw cycles at all PVs without bonus freeze. This concept has been applied by other operators using the CB2 [21], and even a recent report on acute procedural outcomes and short-term follow-up of CB2 ablation with 3-min duration freeze-thaw cycles showed excellent results [22].

In our study, CB2 ablation was associated with significantly shorter procedure (from venous puncture to removal of sheaths) time (74 ± 17 minutes versus 120 ± 49, *p* < 0.05), as compared to CF-RF, which is in line with most of the published comparative studies (RF versus CB) in the literature [11, 19].

Despite AF ablation using cryoenergy and RF ablation with real-time contact force monitoring evolved dramatic improvements, arrhythmic recurrences after the index procedure remain relatively frequent and in most cases are related to PV reconnections [23, 24].

Ciconte et al. reported a significantly higher rate of late PV reconnection following CF-RF ablation when compared with CB2 ablation 9.8–11.7 months after the index procedure. The reported rate of PV reconnection (20.4% versus 36.1%; 1.2 versus 1.8 per patient) and anatomical reconnection pattern around the pulmonary veins were different in the two groups [24]. We found higher reconnection rates both in the CF-RF group (61%, 2.5 per patient) and in the CB2 group (35%, 1.4 per patient) (*p* = 0.01).

In the CB2 group, dominantly the inferior veins reconnected, superior PV reconnection was detected only in 1 out of 7 patients. The explanation is probably the straight orientation of the cryoballoon catheter towards the superior vein antrum, which allows better vessel occlusion, lower nadir temperature, and better tissue-balloon contact, while, in case of difficult occlusion of the inferior PVs, a pull-down technique is needed in order to obtain electrical isolation. Cryoballoon temperatures give reliable information about balloon-tissue contact. Minimum temperature achieved during inferior vein cryoablation was significantly decreased in our study as compared with those measured in the superior PVs.

Although acute isolation can be successfully achieved with CF-RF, late PV reconduction is found in at least one vein during redo procedures [24]. EFFICAS I study has recently showed that 65% of patients following CF-RF procedure experienced a reconnection in at least 1 PV 3 months after ablation, which was related to lower CF values. We found 2.5 nonisolated PVs per patient; the predominant site of PV reconnection was the anterior-inferior segments of the left PVs, as well as the inferior and inferoposterior part of the RIPV. The use of nonsteerable sheath and the longer follow-up (13.3 ± 7.8 months) may be responsible for the different reconnection rates observed in the present study. The lowest FTI values on all left anterior positions are in line with the findings reported in EFFICAS I study, where a deflectable sheath was used in 46% of the patients. The extended use of steerable sheath may result in higher CF values and lower late conduction gaps upon targeting these PV segments.

### 4.1. Periprocedural Complications

No death and no stroke or TIA occurred in our study while pericardial tamponade in one patient (1.7%) in the CF-RF group and phrenic nerve palsy in 3 patients in the CB2 group (7.5%) were observed. The tamponade was successfully treated by pericardial drainage. Luckily, 2 phrenic nerve palsies recovered spontaneously before hospital discharge, and one recovered after 1 year follow-up; our results were similar to previously published data [19].

## 5. Conclusion

Both the second-generation cryoballoon and the contact force RF ablation appeared safe. PVI for paroxysmal AF is faster with CB and results in a similar single procedure success rate at 2-year follow-up as the conventional point-by-point CF-RF.

## Figures and Tables

**Table 1:** Patients' baseline characteristics (LA: left atrial, NOACs: novel oral anticoagulants).

Patients (*n*)	RG group (58)	CB group (40)	*p* value
Age (years)	61 ± 9	59 ± 10	ns
Female gender *n* (%)	20 (34)	13 (32.5)	ns
LA size (mm)	42.1 ± 4.6	41.3 ± 4	ns
Hypertension (%)	30 (51%)	17 (42.5)	ns
Type II diabetes (%)	3 (5.1)	2 (5)	ns
Coronary artery disease (%)	7 (12)	5 (12.5)	ns
Medication prior to ablation			
Beta blockers (%)	55 (95)	40 (100)	ns
Propafenone (%)	24 (41)	12 (30)	ns
Sotalol (%)	2 (3)	0 (0)	ns
Amiodarone (%)	2 (3)	1 (2.5)	ns
Acenocoumarol (%)	40 (69)	29 (72.5)	ns
NOACs (%)	5 (8.6)	4 (10)	ns
